# Myosin VI controls localization of golgi satellites at active presynaptic boutons

**DOI:** 10.1007/s00018-025-05896-2

**Published:** 2025-10-21

**Authors:** Nathalie Hertrich, Yuhao Han, Nathanael Doil, Dimitra Ranti, Britta Eickholt, Anja Konietzny, Marina Mikhaylova

**Affiliations:** 1 RG Optobiology, Institute of Biology, Humboldt-Universität zu Berlin, 10115 Berlin, Germany; 2 Department of Biochemistry, Charité – Universitätsmedizin Berlin, 10117 Berlin, Germany; 3 Institute of Industrial Science, The University of Tokyo, 153-8505 Tokyo, Japan

**Keywords:** Golgi satellites, Myosin VI, Organelle transport, Axon, Synapse, glycosylation

## Abstract

Neurons, as long-lived non-dividing cells with complex morphology, depend on a highly elaborate secretory trafficking system which enables a constant turnover of proteins and membranes. Previously, it was shown that simplified, Golgi-related structures called Golgi satellites (GS) are present in the dendrites of primary hippocampal neurons. These organelles are distinct from the somatic Golgi complex and are involved in *de novo* glycosylation and local forward trafficking of membrane proteins. However, the question of whether GS are also targeted to the axons of principal neurons remained unanswered. In this study, we investigated the subcellular distribution of GS in adult hippocampal neurons. Our findings showed that GS are present all along the axon, extending to the distal tips of the growth cone. Similar to dendritic GS, the axonal organelles are labeled by the same GS markers and are capable of mature glycosylation. Live imaging experiments revealed the presence of both mobile and immobile GS in the axon, and that the switch between active transport and stalling of GS was modulated by neuronal firing. We found that GS frequently pause at *en passant* synapses and remain stationary for longer time periods at activated pre-synaptic boutons. This behavior is dependent on the actin cytoskeleton and the actin-based motor protein myosin VI. Overall, our study demonstrates that neuronal activity can dynamically regulate the positioning of GS in the axon, shedding light on the intricate mechanisms underlying organelle trafficking in neurons.

**Supplementary Information:**

The online version contains supplementary material available at 10.1007/s00018-025-05896-2.

## Introduction

Neurons are highly polarized cells with distinct axonal and somatodendritic domains separated by the pre-axonal exclusion zone and the axon initial segment (AIS). As long-lived, non-dividing cells with a complex morphology and high metabolic demands, neurons depend on the constant delivery, removal and recycling of proteins and membranes to and from distal neurites, which is mediated by an elaborate and well-controlled secretory trafficking system that spans the entire cell. Secretory organelles such as the endoplasmic reticulum (ER), Golgi-related compartments, various types of endosomes, autophagosomes, and lysosomes are found in the soma as well as in the dendrite and axon. Their transport and correct localization are essential for neuronal survival. If impaired, this may lead to severe diseases such as lysosomal storage diseases, Alzheimer’s disease and Parkinson’s disease [1, 2].

The Golgi apparatus, consisting of flattened tubules called cisternae, plays a central role in modifying, sorting, and packaging proteins and lipids into vesicles for transport. Proteins and lipids pass through the Golgi complex, entering from its cis-face and exiting through its trans-face. Along the way, they undergo various post-translational modifications such as glycosylation, phosphorylation, removal of mannose residues, and others. In addition to the somatic Golgi complex, stacked tubular Golgi structures, termed Golgi outposts (GO), have been described at dendritic branch points of drosophila and in the apical dendrite of rodent excitatory pyramidal neurons [3, 4]. GO are frequently clustered near ER exit sites (ERES), and are involved in various local functions, such as protein secretion, trafficking, and glycosylation [5–7]. Previously, we and others have identified another Golgi-related organelle, Golgi satellites (GS) [8–10], which are widely distributed in all dendrites of primary hippocampal neurons. In contrast to GO, GS have a more simplified structure. They do not contain cis-Golgi elements and do not form membrane stacks [9]. Like GOs, GS contain glycosylation machinery and are located in close proximity to the ER and ERGIC, forming a local secretory system where proteins can be recycled, and newly synthesized ER proteins can pass through on the way to the plasma membrane. While some transmembrane proteins do not require mature glycosylation to reach the plasma membrane [11], for many other proteins mature glycosylation can serve as a functional switch. The presence of organelles capable of enabling mature glycosylation could obviate the need for a protein to be transported back to the soma. In a recent study, Andres-Alonso et al. (2023) demonstrated that GS membranes exhibit characteristics similar to those of the trans-Golgi network, enabling local mature glycosylation in dendritic segments. Through metabolic labeling, they identified distinct GS populations, each capable of performing various glycosylation processes, including sialylation. Along these lines, another study has suggested that GS are involved in the activity-dependent shaping of the dendritic surface glycoproteome of rat neurons [10].

Are GS present in axons, and can they mediate glycosylation of axonal proteins? In axons, Golgi-related compartments are not well investigated, with only a few descriptive studies reporting on them. Indeed, in the peripheral nervous system, Golgi components are present in axons of the rat sciatic nerve and GS have been described to be involved in trafficking of cold-sensitive TRPM8 ion channels in axons of dorsal-root ganglion neurons [12, 13]. In the central nervous system, while soma-derived trans-Golgi carriers can deliver cargoes (i.e. lysosomal proteases) to the distal axon of cortical neurons, nothing is currently known about the axonal abundance, distribution and trafficking of GS [14]. Previous studies [9, 10] have primarily focused on local protein glycosylation in dendrites. However, some of these glycoproteins can be found in axons. For example, the polysialylated form of neuronal adhesion molecule 1 (PSA-NCAM1), which was investigated by Andrés-Alonso et al. (2023) is also highly abundant in axons. PSA addition modulates the adhesion properties of NCAM1 and is important for axonal differentiation and sprouting [15, 16]. Given the length of axonal compartments and the existence of local protein translation and recycling in distal axons, it is plausible that axonal GS, similar to dendritic GS, could also be responsible for protein glycosylation.

Long-range transport of organelles between the soma, dendrites, and axon is mediated by the microtubule (MT) cytoskeleton and kinesin and dynein motor proteins, whereas short-range transport and anchoring at specific sites rely on the F-actin cytoskeleton and the activity of the myosin motors. Although not previously explored, it is likely that GS undergo MT and F-actin-based transport in both dendrites and axons of mammalian neurons.

Here, we investigated the distribution and trafficking of GS in axons of adult rat hippocampal neurons. We found that axonal GS contain sialyltranferases, are glycosylation-competent and present along the entire axon, including growth cones. Their bidirectional motility and the switch between long-range transport and anchoring at specific sites is regulated by neuronal activity and the motor protein myosin VI.

## Materials and methods

### Animals

Wistar Unilever HsdCpb: WU (Envigo) rats were obtained from the animal facility of the University Medical Center Hamburg-Eppendorf, UKE, Hamburg, Germany, from the animal facility of the Humboldt-Universität zu Berlin and from Janvier laboratories. Protocols for prepping primary neuronal cultures were approved by the local authorities of the city-state Hamburg (Behörde für Gesundheit und Verbraucherschutz, Fachbereich Veterinärwesen), the animal care committee of the University Medical Center Hamburg-Eppendorf as well as the local authorities of the city-state Berlin (Landesamt für Gesundheit und Soziales).

### Key resources, reagents, constructs and cloning

Detailed information about the antibodies, DNA plasmids and pharmacological compounds used in this study can be found in the Appendix Table S1.

All constructs used in this study were verified by sequencing and are summarized in the Appendix Table S1. Restriction enzymes were purchased from Thermo Fisher Scientific.

The St3gal5-GFP plasmid was cloned by PCR amplification of the St3gal5-GFP [8] and inserting it into a pAAV backbone using EcoRI and HindIII restriction sites. The pORANGE_Actin was produced by exchanging the GFP in the Addgene #139,666 vector (pORANGE_Actin#2-GFP) for a TagRFP (amplified from pTagRFP-C, Evrogen) using the restriction enzymes NheI and BamHI. The St3gal5-Halo was produced by exchanging the GFP sequence in the St3gal5-GFP vector for a HaloTag sequence, amplified from the template pHTN-HaloTag (gift from Andrew Plested, Humboldt-Universität zu Berlin) using the restriction enzymes BamHI and HindIII. The myosin VI full length was produced by subcloning the myosin VI full length from pL7-mGFP (gift from Wolfgang Wagner) into a pmEmerald-C1 vector.

### Primary rat hippocampal cultures and transfections

Primary hippocampal rat cultures were prepared as described previously [17] with minor modifications. Briefly, brains from E18/19 embryos or P0 pups were collected and the hippocampi were dissected and collected in HBSS (Thermo Fisher Scientific). After washing with HBSS, the hippocampi were incubated with trypsin (0.25%, Thermo Fisher Scientific) for 10 min at 37 °C and then dissociated with a syringe (first 0.9 mm, then 0.45 mm). The dissociated cells were plated on poly-L-lysin (Sigma-Aldrich) coated coverslips at a density of 40.000–60.000 cells/mL in full medium (Dulbecco’s modified Eagle’s medium (DMEM; Gibco, Thermo Fisher), 10% fetal calf serum (FCS) (Gibco), 1× penicillin/streptomycin (Thermo Fisher Scientific), and 2 mM glutamine (Thermo Fisher Scientific). After 1 h the medium was exchanged to BrainPhys neuronal medium (STEMCELL Technologies) with SM1 supplement (STEMCELL Technologies) and 0.5 mM glutamine. Cells were incubated at 37 °C, 5% CO2 and 95% humidity.

Primary rat hippocampal cultures were transfected with Lipofectamine 2000 (Thermo Fisher Scientific) at a ratio of 1:2 DNA: Lipofectamine 2000 at DIV 12–16 according to the manufacturer’s instructions. Experiments were performed 24 to 48 h after transfection. For the transfection, the conditioned medium was exchanged with BrainPhys (without supplements). After 1.5 h incubation with the transfection mixture, the medium was replaced again with the prewarmed conditioned medium.

### Immunocytochemistry (ICC)

For immunocytochemistry, cells were fixed with 4% Roti-Histofix (Carl Roth)/4% sucrose for 10 min at room temperature (RT) and washed 3 times with PBS. The cells were then permeabilized (0.2%Triton X-100 (Supelco) in PBS) for 10 min, washed 2x with PBS and blocked with BBHE (BBHE: 0.1% TritonX, 10% heat-inactivated horse serum (Gibco), in PBS) for one hour at RT. When cells were transduced with St3gal5-GFP virus the signal was enhanced with an Atto488 pre-labeled GFP nanobody (Table S1). Primary antibodies were added in BBHE overnight at 4 °C. Cells were washed again with PBS 3x before secondary antibodies were added in BBHE for 1 h at RT. Finally, the coverslips were washed 3 times with PBS and then mounted in mowiol (prepared according to the manufacturer’s instructions: 9.6 g mowiol 4‐88 (Carl Roth), 24.0 g glycerin, 24 ml H_2_O, 48 ml 0.2 M Tris pH 8.5, including 2.5 g DABCO (Sigma‐Aldrich)) on microscope slides before imaging.

### AAV production and transduction of primary hippocampal neurons

Adeno-associated viruses 9 (AAV9) were produced at the vector facility of the University Medical Center Hamburg-Eppendorf (UKE). Primary rat hippocampal cultures were transduced at DIV 16–17 with AAV9 carrying the synapsin-St3gal5-GFP vector (1 µl virus per 18 mm coverslip, viral genomes per µl = 4.6-5.6E + 13 vg/µl) in conditioned medium. After 4–5 days, at DIV 20–21, the cells were fixed (see above) and used for further experiments.

### Cell lines and transfection of cell lines

HEK293T cells were cultured in full medium containing Dulbecco’s modified Eagle’s medium (DMEM; GIBCO, Thermo Fisher) and 10% fetal calf serum (FCS), 1× penicillin/streptomycin, and 2 mM glutamine at 37 °C, 5% CO_2_, and 95% humidity. Cells were transfected with MaxPEI (Polysciences 24765-1) at 70% confluency. The DNA MaxPEI ratio was 1:3 and the transfection performed according to the manufacturer’s instructions. The cells were harvested 24–36 h after transfection.

### Co-immunoprecipitation and Immunoblotting

For co-immunoprecipitation, HEK293T cells grown in 10 cm dishes were transfected with a myosin VI dominant negative fused to EGFP or just with EGFP alone as a control. Cells were harvested 24–36 h after transfection. For the harvest, the cells were first washed with 4 °C TBS (20 mM Tris HCl (pH 7.4), 150 mM NaCl, 0.1% TritonX-100). Afterwards, cells from one 10 cm dish were lysed with 400 µl lysis buffer (50 mM HEPES pH 7.4, 300 mM NaCl, 0.5% Triton X-100, complete protease inhibitor cocktail [Roche]). The lysate was centrifuged at 13.000 x g for 15 min and the supernatant (SN) was collected. At this stage, a sample of the SN was taken as “input”. The remainder of the SN was incubated with magnetic GFP- trap beads (Chromotek) on a rotor over night at 4 °C. The beads were then washed 5x with lysis buffer (without protease inhibitor) using a magnetic rack before all lysis buffer was removed and the beads were resuspended in 80 µl 4x SDS buffer. The samples were then denatured at 98 °C for 10 min and subjected to SDS-PAGE (10%) and immunoblotting. For the detection of myosin VI (full-length and dominant negative), the membrane was first developed with myosin VI antibody (Table S1) and in a second step incubated with GFP antibody (Abcam, Table S1) and developed a second time. The secondary antibody was ms-HRP-coupled and detected with SuperSignal™ West Pico PLUS Chemiluminescent Substrate (Thermo Fisher Scientific).

### Pharmacological treatments

#### Latrunculin A (LatA) treatment

For the LatA treatment experiments, cells were incubated with a final concentration of 5 µM latrunculin A (2 mM stock in DMSO, Table S1) in prewarmed conditioned medium or aCSF (145 mM NaCl, 10 mM HEPES, 12.5 mM D-glucose, 1.25 mM NaH_2_PO_4_, 2.5 mM KCl, 1 mM MgCl_2_, 2 mM CaCl_2_, pH adjusted to 7.4) for 30 min at 37 °C, 5% CO2 in 95% Humidity. After 30min incubation, the cells were imaged for a maximum of 45 min. In the control group, cells were treated with DMSO (Sigma-Aldrich).

#### Synaptotagmin antibody uptake assay

For the synaptotagmin antibody uptake assay, cells were incubated with pre-labeled synaptotagmin antibody (Table S1) (1:100) in aCSF for 30 min at 37 °C, 5% CO2 in 95% Humidity. After 30min, the medium was exchanged to antibody-free medium for live imaging.

#### HaloTag labeling

When HaloTag constructs were used, the conditioned medium was removed from the transfected cells and replaced with aCSF (see LatA treatment) containing the HaloTag dye (Promega, according to manufacturer’s instructions, Table S1). After 30 min of incubation the medium containing the HaloTag dye was replaced with the conditioned medium and cells were incubated again for at least 30 min before imaging.

### Click chemistry for visualizing de Novo glycosylation

For metabolic labeling of primary hippocampal neurons, cells were first transfected with St3gal5-Halo and mRuby as a morphological marker and then the sugars Ac4GalNAz (1µM), Ac4GlcNAz (1µM) or a DMSO control were directly added for 24 h. The cells were then treated with the HaloTag-dye JF676 and 1µM DBCO-AF488 to label the sugars for 40 min at 37 °C and 5% CO2. Afterwards the cells were quickly washed 3 times with warm PBS, fixed with 4% PFA for 10 min, washed in PBS and mounted with Mowiol on imaging slides.

### Wide-field, total internal reflection fluorescence (TIRF), and confocal spinning‐disk microscopy

Live-cell microscopy and fixed cell imaging were performed with a Visitron Spinning-Disc TIRF-FRAP system on a Nikon Eclipse Ti-E controlled by VisiView software (Visitron Systems). Samples were kept in focus with the built‐in Nikon perfect-focus system. The fluorophores were excited by 405, 488, 561 and 640 nm laser lines, coupled to the microscope via an optic fiber. The samples were imaged (confocal and TIRF) with a 100x TIRF oil objective (Nikon, ApoTIRF 100×/1.49 oil) resulting in images with a pixel size of 0.065 μm. Oblique and TIRF illuminations were obtained with an ILAS2 (Gattaca systems) TIRF system. Multichannel images were acquired sequentially or simultaneously (TwinCam, Cairn Research) using an appropriate filter set with an Orca flash 4.0LT CMOS camera (Hamamatsu). Confocal spinning disc imaging was performed with the Yokogawa CSU-X1 unit. For fixed cell imaging, multichannel z-stacks (distance 0.3 μm) were taken sequentially using an appropriate filter set with an Orca flash 4.0LT CMOS camera (Hamamatsu) or a pco.edge 4.2 bi sCMOS camera (Excelitas PCO GmbH). For live-cell imaging experiments, the images were acquired at 2 frames per second (single focal plane) or at specified indicated intervals. The samples were incubated in a stage top incubator (Okolab) at 37 °C, 5% CO2 and 90% humidity atmosphere. Neurons were imaged in regular culture medium unless specified otherwise. Coverslips were placed in a Ludin chamber (Life Imaging Services). If pharmacological treatment was performed during live imaging, the respective agent was added manually to the culture medium and incubated for the indicated time spans in the top stage incubator.

#### Calcium imaging

For the calcium imaging experiments, primary hippocampal neurons DIV 14–16 were co-transfected with St3gal5-Halo and GCaMP6s for 24 h. Before imaging, the HaloTag was labeled with JF646 (Promega) or JF549 (Promega) as described above. The cells were imaged with the TIRF dual-cam system simultaneously with 10 FPS for 3 min.

### Experimental design and statistical analyses

The researcher performing data analysis was blinded to the identity of the experimental groups.

#### Kymograph analysis

Kymographs were created with the Fiji plugin from KymographClear/or KymographClear2a [18]. For each cell, one axonal stretch was selected using the segmented line tool, and the line thickness was chosen to cover the axon thickness. For analyses of retrograde and anterograde events, kymographs were created with the segmented line starting from the cell body. GS trajectories shown in the kymograph were traced by hand using the straight-line tool. Moving trajectories (= slope) were traced separately from pausing/stationary events (= vertical lines). From the angle and length of the trajectories, the instant run length, the instant velocities and the instant pausing times were calculated. Times during which the GS did not move were divided into “pausing” (less than 60 s without processive movement) and “stationary” (more than 60 s without processive movement). To count pausing events and to localize them in the kymograph, the pointing tool was used to measure x/y values.

#### Analysis of fluorescence intensity of mobile and immobile GS

The same axonal ROIs selected for kymograph analysis (see above) were used to quantify the fluorescence intensity of mobile and stationary GS. GS moving through these ROIs were tracked using the FIJI plugin *TrackMate* [19]. To distinguish between mobile and immobile GS, tracking was performed twice for each ROI using different parameter settings. For all image stacks, the LoG detector was used with an object diameter set to 0.45 μm. The quality threshold was manually adjusted for each stack to account for variations in signal-to-noise ratio and to ensure accurate GS detection.

For stationary GS, the following parameters were used:

- *Simple LAP tracker*: Linking max distance – 1 μm; Gap-closing max distance – 0 μm; Gap-closing max frame gap – 0 μm.
- *Track filters*: Track displacement < 2 μm; Track duration > 60 frames.

For mobile GS, the parameters were adjusted as follows:

- *Simple LAP tracker*: Linking max distance – 4 μm; Gap-closing max distance – 7 μm; Gap-closing max frame gap – 1 μm.
- *Track filters*: Track displacement > 2 μm; Track duration > 4 frames.

Mean fluorescence intensities of the tracked mobile and immobile GS were extracted from the TrackMate summary output, which provides mean fluorescence values for each tracked spot across all frames. The median fluorescence intensity for mobile and immobile GS were then compared within each axon.

#### Analysis of pausing/stationary events at presynaptic boutons

To analyze pausing and stationary events at presynaptic boutons, axons were traced, and boutons were selected according to their morphology with the FIJI segmented line tool in the timelapse image stack. The kymograph Distances between boutons were calculated using the Fiji macro MeasureSegementedDistances (10.6084/m9.figshare.1133868.v1). Active boutons were distinguished from inactive boutons by live labeling with synaptotagmin antibodies. The distances between (active/inactive) boutons, measured in the timelapse videos, were used to determine the position of (active/inactive) boutons (x value) along the corresponding kymograph. To calculate the distance of the pausing/stationary event (x value of the event) to the nearest bouton, the distance to all boutons was calculated and the smallest was selected. If the event occurred within 1 μm (distance) to the next bouton it was counted as pausing/being stationary at an (active/inactive) bouton. For an intrinsic control, random pausing events of the same number and in the same range to the corresponding kymograph were created in Excel. These random events were then treated in the same way as the “real” values to calculate the random pausing/being stationary % values. For statistics, a paired t-test was performed using Prism 9.1 (GraphPad).

#### Calcium imaging analysis

To analyse the % of active boutons that contained a stationary GS and the % of stationary GS being at active boutons, kymographs were created along the axons imaged as described above using the kymograph builder plugin of Fiji. First, using the multiple point tool all the stationary GS (being immobile for at least 60 s) were selected and their x/y coordinates exported. Next, the locations of GCaMP6s fluorescence intensity spikes were selected and their x/y coordinates exported in the same way. As described above the distance of the stationary event (x value of the event) to all the boutons was calculated in excel and the smallest was selected. If the event occurred within 1 μm (distance) to the next bouton it was counted as being stationary at an active bouton. For an intrinsic control, random stationary events of the same number and in the same range to the corresponding kymograph were created in excel. These random events were then treated in the same way as the “real” values to calculate the random being stationary % values. For statistics, a paired t-test was performed using Prism 9.1 (GraphPad).

#### GS counting

To count GS, primary hippocampal neurons were transduced or transfected with St3gal5 and fixed as described above. To distinguish the axon from the dendrite, ICC was performed and the AIS (ankyrin G) or presynaptic (bassoon) or somatodendritic compartment (MAP2) was labeled. The stained and mounted coverslips were imaged using a spinning disc confocal microscope. To enlarge the field of view, multiple images were taken using the scan slide tool and the resulting images were then stitched together using a Fiji macro. The images were then analyzed using Fiji. The axon was divided into three compartments: the proximal part (~ 50–100 μm distance to the soma), the medial part (~ 150–200 μm distance to the soma) and the distal part (at least ~ 250 μm distance to the soma). The segmented line tool was used to measure the distance to the soma. Within each compartment, if possible, a stretch of axon was selected (proximal, medial: 40–72 μm; distal: 40–95 μm) and the GS vesicles were detected using the “Find Maxima” command with the threshold manually adjusted. The number of GS was then counted and normalized to 10 μm for each axonal area.

#### Co-localization analysis of GS and myosin VI

To analyze co-localization of GS and the full-length myosin VI (MyoVI-FL) in axons, primary hippocampal neurons were co-transfected with St3gal5-Halo and MyoVI-FL constructs. Transfected neurons were pre-labelled with a Halo ligand as described above, then fixed, labeled with MAP2 antibodies and imaged using a spinning disk confocal microscope with 100x oil objective. Z-stacks were taken with a 0.2 μm step size. The images were analyzed with FIJI. GS in the medial part (~ 150–200 μm distance to the soma) or the distal part (at least ~ 250 μm distance to the soma) of the axon were detected by finding the maximum fluorescent intensity of the St3gal5 channel. The detected ROIs representing GS were then projected to the MyoVI-FL channel. MyoVI-FL clusters within 0.26 μm radius of the GS were counted as co-localized myosin VI.

#### Co-localization analysis of GS and pGolt in axon

For the investigation of GS and pGolt co-localization, primary hippocampal neurons were co-transfected with constructs expressing St3gal5 and pGolt. Transfected neurons were then imaged under live condition on spinning disk confocal microscope with 100x oil objective which results in a pixel size of 0.069 μm. For the colocalization analyses the first frame of the image was selected and the images were then analyzed using Fiji. The colocalization analysis of the GS at the axons was performed with the plug-in tool ComDetv 0.5.0. The puncta were detected by the pixel size ranging from 3 to 4, as well as the intensity ~ 20, depending on the transfection efficiency. Detection of larger puncta and segmentation was also selected. The counted particles were normalized with the selected area and the percentage of colocalization was measured.

#### Volume analyses

To measure the volume of the GS, mature primary hippocampal neurons were transfected with St3gal5-Halo and MyoVI-dn-GFP or GFP as a control fixed as described above. To distinguish the axon from the dendrite, ICC was performed and the somatodendritic compartment (MAP2) was labeled. The stained and mounted coverslips were imaged with a z-stack (0.2 μm step size) using a spinning disc confocal microscope. To enlarge the field of view, multiple images were taken using the scan slide tool and the resulting images were then stitched together using a Fiji macro. The images were then analyzed using Fiji. The medial part (~ 150–200 μm distance to the soma) of the axons was chosen for analyzing the volume of the GS. The segmented line tool was used to measure the distance to the soma. Within the medial part, a stretch of axon was selected. For the dendrites, the same process was followed, and the area chosen for quantification was at least ~ 50 μm away from the soma. The analysis was performed with the Image J plug-in 3D objects counters, counting the number of 3D objects in a stack, displaying the volume. The Data is represented as median per cell or in a histogram of all cells, binned in 0.01 µm^3^ steps.

## Statistics

Statistical analyses were performed using Prism 9.1 (GraphPad), with detailed specifications of tests, significance levels, n numbers, and biological replicates included in the figure legends. Data are presented as individual dot plots, and error bars indicate means with their standard deviation across the manuscript. Statistical significance is defined as: n.s. for not significant, * for *p* < 0.05, ** for *p* < 0.01, *** for *p* < 0.001, **** for < 0.0001.

For the kymograph analyses, run lengths and velocities are measured as the median per cell and presented as mean value for all cells. The pausing time is measured as average per cell and presented as mean value for all cells.

## Results

### GS are abundant in axons and involved in de Novo protein glycosylation

**Fig. 1:**
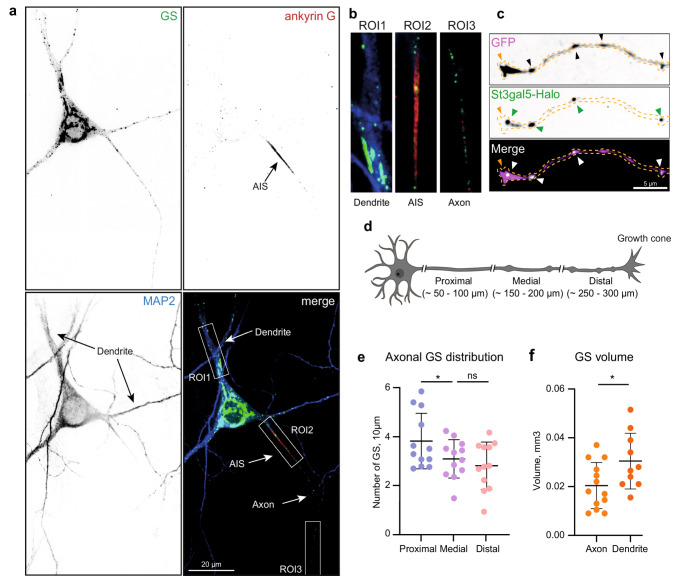
Distribution of GS in axons. **a**) Representative image of GS (green) in dendrites (region of interest 1; ROI1), in the AIS (ROI2) and in the proximal axon (ROI3) of primary hippocampal rat neurons (DIV16-17), stained with ankyrin G as an axon initial segment marker and MAP2 as a dendritic marker. GS were labeled with St3gal5-GFP and the signal intensity was boosted with an Atto488 prelabeled GFP nanobody. **b**) ROIs 1–3 highlighted in a), showing GS (green) in the dendrite (left panel), AIS (middle panel) and in the axon (right panel). **c**) Representative image of an axonal neuronal growth cone labeled with St3gal5-HaloTag JF646 and eGFP, Upper: highlighting the boutons (black arrows) and the growth cone (orange arrow); middle panel: highlighting GS (green arrows); lower panel: merge. **d**) Schematic showing the axonal areas used for quantifying GS abundance in e Quantification of GS distribution within the axon. Values are normalized to GS per 10 μm axon and show a significant difference for proximal area to the medial and no difference for the medial to the distal area (p* (proximal-medial) = 0.0405, p (medial-distal) = 0.4649; repeated measurements ANOVA test; *n* = 12). Values are presented as mean ± SD. **f**) Quantification of the volume of GS in the axon and in the dendrite, showing that dendritic GS are significantly bigger than axonal GS (p* = 0.0316); unpaired t-test, n: (axon-ctr) = 13, (dendrite-ctr) = 11; one axon/dendrite per cell in two individual cultures; single values represent the median per cell, presented as mean ± SD

**Fig. 2:**
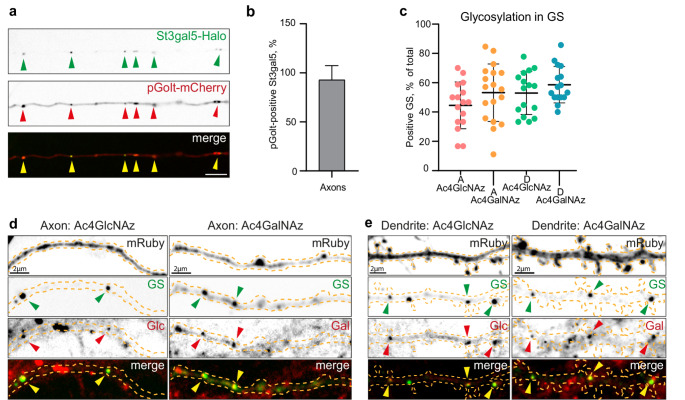
Functional GS in axons. **a**) Confocal image of the axon containing GS co-labeled with St3gal5 (upper) and pGolt (middle). Colocalization of both markers is shown in the merge image (lower). **b**) Quantification of the colocalization of St3gal5 and pGolt shown in d), mean = 90%. The values are presented as percent of all St3gal5 labelled GS positive for pGolt, *n* = 53 axons in 3 independent cultures. **c**) Quantification of GS positive for Azide-functionalized glycoconjugates (Ac4GlcNAz and Ac4GalNAz) in axons (A) and dendrites (D) via metabolic labeling. Axon: mean (Ac4GlcNAz) = 44.56%, mean (Ac4GalNAz) = 53.21%; Dendrite: mean (Ac4GlcNAz) = 52.97%, mean (Ac4GalNAz) = 58.63%. The values are presented as percent of all GS; n (axon, Glc/Gal) = 16/18; n (dendrite, Glc/Gal) = 16/16; one axon or dendrite per cell; 2 independent cultures. **d**) Representative confocal images of axons of primary hippocampal neurons transfected with St3gal5-Halo (dye: JF646) and mRuby as morphological marker. Neurons are metabolically labelled with Ac4GalNAz or Ac4GlcNAz. Glycans visualized with live staining using DBCO-488 probe. **e**) Representative confocal images of dendrites of primary hippocampal neurons transfected with St3gal5-Halo (dye: JF646) and mRuby as morphological marker. Neurons are metabolically labelled with Ac4GalNAz or Ac4GlcNAz. Glycans visualized with live staining using DBCO-488 probe

GS have been described and characterized previously in dendrites of hippocampal primary neurons. However, the presence of axonal GS has only been reported in the axons of peripheral neurons [8–10, 12]. During earlier work, we observed vesicles that were positive for GS markers in axons. We therefore set out to perform a detailed follow up investigation of the distribution, localization, and trafficking of GS in axons of rat hippocampal primary neurons. To visualize GS, we transduced or transfected dissociated primary neurons with the GFP- or HaloTag (Halo) coupled ST3 β-galactoside α−2,3-sialyltransferase 5 (St3gal5), a tool previously described as a marker of GS and other Golgi-related compartments (Mikhaylova et al., 2016; Andres-Alonso et al., 2023). To identify neuronal subdomains, we performed immunocytochemistry (ICC) in adult neurons (DIV21-23) expressing tagged St3gal5 and stained for ankyrin G, an AIS marker, and MAP2, a somatodendritic marker. Indeed, confocal images showed that St3gal5 labeled the somatic Golgi complex, dendritic Golgi outposts, GS in dendrites, as well as GS in the AIS, in the axon (Fig. 1a-b), and in the axonal growth cone (Fig. 1c). To characterize the axonal distribution of GS, we subdivided the axon into three areas: the proximal part (~ 50–100 μm distance to the soma), the medial part (~ 150–200 μm distance to the soma), and the distal part (at least ~ 250 μm distance to the soma) (Fig. 1d). The number of GS per 10 μm was quantified in the three areas, revealing that there are slightly more GS in the proximal region of the axon compared to the medial or distal part (mean: proximal = 3.8 per 10 μm, medial = 3.1 per 10 μm, distal = 2.8 per 10 μm) (Fig. 1e). We next analyzed the relative volume of GS and found that axonal GS were smaller than dendritic (mean volume axon: 0.02 µm^3^; dendrite: 0.03 µm^3^ (Fig. 1f). Expanding on the findings of a previous study, which demonstrated that St3gal5 and another well-characterized GS marker pGolt label the same structures in dendrites [8], we also observed a high degree of colocalization between these markers in axons (Fig. 2a-b) (mean = 90%). In comparison to pGolt, St3Gal5 showed less membrane labeling and a higher signal-to-noise ratio. Additionally, endogenous St3Gal5 is highly expressed in the brain, and expressing tagged St3Gal5 can reflect the localization of the endogenous protein. Therefore, we decided to use tagged St3gal5 as the GS marker for further analysis.

To investigate whether, in analogy to dendrites, GS in the axon contain a functional glycosylation machinery, we employed click chemistry to label locally incorporated azido monosaccharides. Neurons expressing St3gal5-Halo were incubated for 24 h with tetraacetylated N-azidoacetyl-glucosamine (Ac4GlcNAz) and N-azidoacetyl-galactosamine (Ac4GalNAz) to predominantly label N- and O-linked glycans, respectively. Subsequently, St3gal5-Halo was labeled with JF646, while the sugars were labeled with DBCO-AF488 (Fig. 2d-e). Analysis of confocal images indicated that a large proportion of St3gal-5 labeled GS in dendrites were positive for Ac4GlcNAz (52,97%) and Ac4GalNAz (58,63%) (Fig. 2c), which align with the findings of Andres-Alonso et al. [9]. Quantifications have shown that in axons 44,56% of GS were positive for Ac4GlcNAz and 53,21% for Ac4GalNAz (Fig. 2c), which is in a similar range as in dendrites. These findings collectively suggest that axonal GS are capable of protein glycosylation.

### Mobile and immobile GS are present in axons

**Fig. 3:**
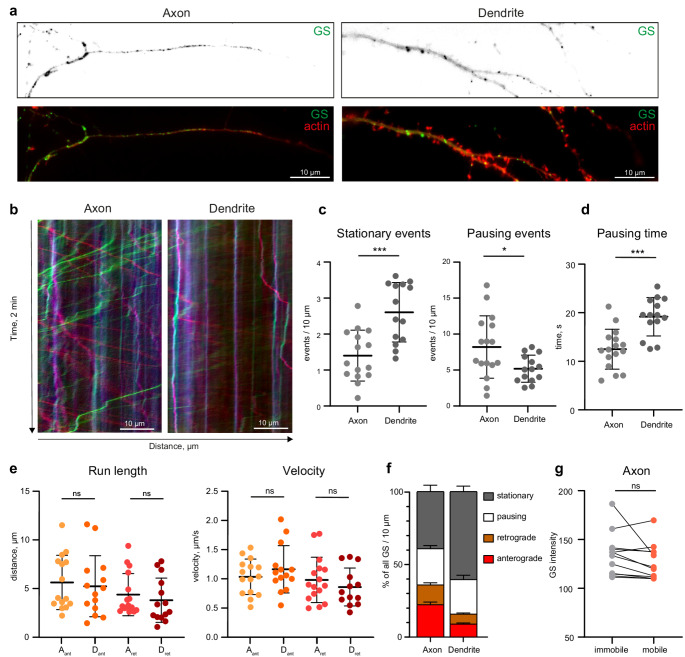
Trafficking of GS in the proximal axon. **a**) Representative confocal images of axons (left) and dendrites (right) of neurons expressing the GS-marker St3Gal5-GFP and actin-RFP. See also videos 1 (dendrite) and 2 (axon). **b**) Kymographs of the videos shown in A), imaged for 2 min at 4 frames per second (FPS), showing higher mobility of axonal GS compared to dendritic GS. **c**) Quantification of stationary and pausing events per 10 μm showing a highly significantly higher amount of stationary events (not moving for at least one minute) in dendrites than in axons (p*** = 0.0002; unpaired t-test; n (axon) = 15, n (dendrite) = 14; one axon or dendrite from one cell in 3 individual cultures), whereas the number of pausing events (not moving for less than 60 s) was significantly reduced in dendrites (p* = 0.0224; unpaired t-test), presented as mean ± SD. **d**) Quantification of the GS pausing time in axons and in dendrites, which is significantly longer in dendrites (p*** = 0.0001; unpaired t-test, same n as in c). Values are presented as mean ± SD. **e**) Quantification of GS trafficking parameters: run length and velocity (divided in anterograde movement (Ant) and retrograde movement (Ret) in Axons (A) and in dendrites (D). The trafficking parameters are not significantly different (p (anterograde run length) = 0.7222, p (retrograde run length) = 0.4777, p (anterograde velocity) = 0.3406, p (retrograde velocity) = 0.3765; unpaired t-test; same n as in c) Values are presented as mean ± SD. **f**) Percental distribution of GS motility states in axons and dendrites: being stationary, pausing, being transported retrogradely or anterogradely displayed as % of the time the GS spend in that stage. Note a longer stationary time in dendrites as well as less retrograde and anterograde transpor. **g**) Quantification of the fluorescence intensity of mobile and immobile GS in the axon (*p* = 0.0961); paired t-test, *n* = 12 axons in 3 individual cultures; single values represent the median per cell, presented as mean ± SD

As previously reported, GS are not only stationary but can be actively transported bidirectionally in dendrites [8]. To investigate the mobility of GS in axons, we performed live imaging of primary hippocampal neurons co-transfected with St3gal5-GFP, to visualize GS, and with actin-RFP, which outlines the neuronal membrane and is enriched in both presynaptic boutons and dendritic spines, allowing for the identification of subcellular compartments (Fig. 3a, video 1–2).

To compare GS trafficking in axons and dendrites, we analyzed kymographs from time-lapse imaging series (Fig. 3b). Similar to dendrites, both immobile and bidirectionally moving GS were observed in the axon. We decided to separately investigate four mobility parameters [20]: being stationary (immobile for a minimum of 60 s), pausing (immobile for less than 60 s), retrogradely transported or anterogradely transported. Although the stationary events (Fig. 3c) occurred more frequently in the dendrites, the number of pausing events was lower in the dendrite (mean (stationary) = 2.5 per 10 μm; mean (pausing) = 6.4 per 10 μm) compared to the axon (mean (stationary) = 1.4 per 10 μm; mean (pausing) = 10.1 per 10 μm). However, the pausing times (Fig. 3d) were significantly longer in dendrites (mean = 19.1 s) than in axons (mean = 12.5 s). The average run length and velocity did not differ significantly between the compartments (Fig. 3e). Our findings indicate that GS exhibit considerably greater mobility in axons compared to dendrites (~ 35% vs. ~15% mobile, Fig. 3f). To investigate if there is difference between mobile and immobile GS populations, we examined the fluorescence intensity of the GS as a proxy for their size. We observed that the mobile GS exhibited a slightly lower, though not significant, intensity compared to the immobile GS, (Fig. 3g), indicating that all types of GS can be stationary.

### GS pause at En passant synapses and are anchored to active boutons

**Fig. 4:**
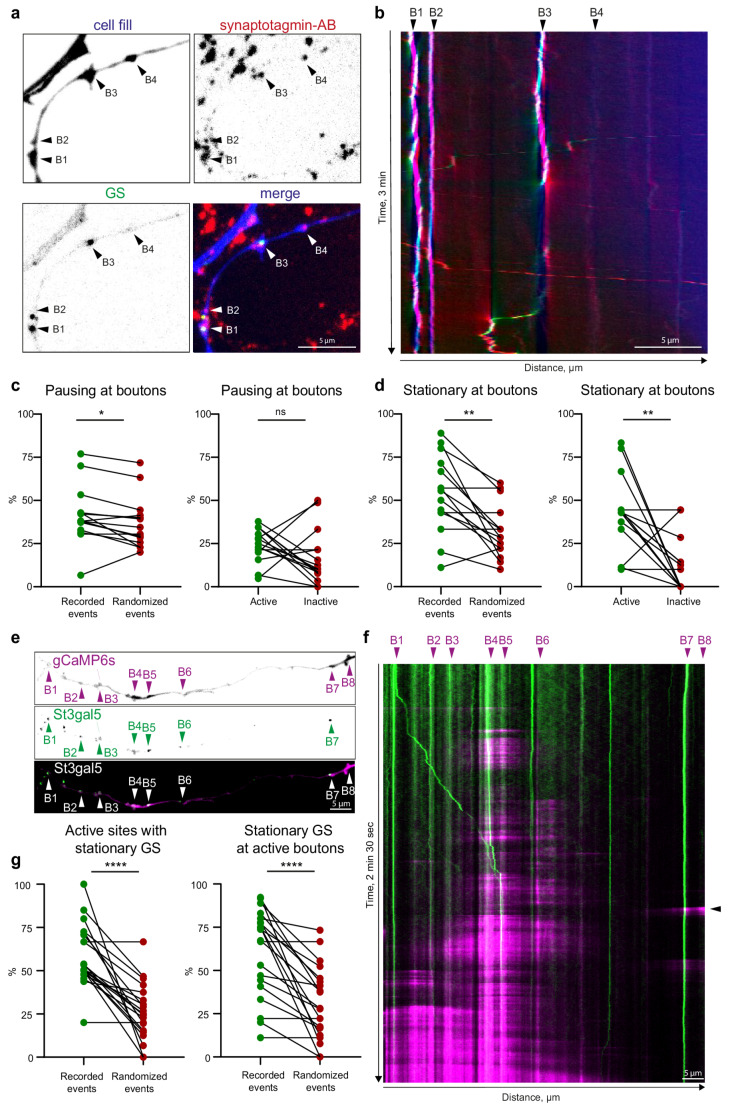
GS pause at presynaptic boutons and are anchored to active boutons. **a**) A representative image showing GS (green) at active presynaptic boutons (red) in DIV16-17 hippocampal neurons. Black arrows pointing to active boutons (B1-4); active boutons are labelled via synaptotagmin antibody uptake assay, also see video 3. **b**) Representative kymograph of the axon (shown in a) with red arrows pointing to the active boutons defined in A: (B1-4), showing multiple stationary events at active boutons. **c**) Description of GS pausing at boutons (left) but independent of bouton activity (right). For intrinsic control, GS pausing events are compared to random pausing events distributed along the examined axon (see Methods), (p* (GS-random) = 0.0199, p* (active-inactive) = 0.1716; paired t-test; *n* = 15, from three independent cultures). **d**) GS are stationary at boutons (left) and preferentially stationary at active boutons (right), same method and n as c) (p** (GS-random) = 0.0012, p** (active-inactive) = 0.0017; paired t-test). **e**) Representative confocal images of GS (upper panel, green) with a calcium indicator GCaMP6s (middle panel, magenta) and a merge; arrows indicate GS (green) and boutons showing calcium transients (magenta). **f**) Representative kymograph of the cell shown in e), with black arrows highlighting the boutons showing calcium transients and the magenta arrow showing the represented frame in e. Axons were imaged for 3 min, with 10 frames per second. **g**) Quantification of the calcium imaging represented in f, showing that flashing boutons colocalize (+−1 μm) frequently with a stationary GS which is highly significant compared to a randomized distribution of GS along the same axonal stretch (p**** = <0.0001; paired t-test). Stationary GS frequently colocalize with boutons undergoing activity; values are compared to a randomized distribution of GS (p**** = <0.001; paired t-test), *n* = 20 axons in 2 independent cultures

As we observed both mobile and immobile GS in axons, we next asked whether mobile GS stop randomly, or at specific locations. Given that other organelles, such as amphisomes and dense-core vesicles (DCV), are known to stall at presynaptic boutons [21, 22], we sought to determine whether immobile GS localize preferentially to presynaptic sites and whether this correlates with synaptic activity at the bouton. To investigate this, we co-transfected primary hippocampal neurons with St3Gal5-GFP and with a cell fill (mRuby) to morphologically visualize presynaptic boutons. Subsequently, we performed time-lapse imaging in combination with a synaptotagmin antibody uptake assay to label active boutons (Fig. 4a, Video 3).

Images were taken in the medial/distal region of the axon, given that synapses are more frequent in this part of the axon [22, 23]. To analyze GS trafficking, kymographs were created (Fig. 4b) and pausing and stationary events were identified. We further examined whether these events occurred at active or inactive synaptic boutons, defined as those within ± 1 μm of the bouton center, based on the work by Guedes-Dias et al. [24]. As an intrinsic control, we compared these results to the same events after they have been randomized along the kymograph (see Material & Methods). We found that short-term pausing events occurred at a significantly higher frequency at boutons than would be expected from random pausing events (Fig. 4c, left), irrespective of their activity status (Fig. 4c, right). Notably, stationary GS were not only found preferentially at boutons (Fig. 4d, left), but specifically at active synapses (Fig. 4d, right). To investigate the influence of neuronal activity in a more direct manner we performed simultaneous imaging of calcium indicator GCaMP6s and St3gal5-Halo (Fig. 4e). We observed flashes of calcium which report periods of activity at boutons. We created kymographs (Fig. 4f) and analyzed the percentage of instantly active boutons containing a GS (being stationary within +- 1 μm) and the percentage of GS being stationary at an active bouton (Fig. 4g). Both types of analysis showed a significant colocalization between the GS and an activity indicator compared to a random distribution of stationary GS within the same stretch of axon. Taken together, this indicates that GS are recruited to boutons in an activity-dependent manner.

### The axonal F-actin network disrupts processive trafficking of GS

**Fig. 5:**
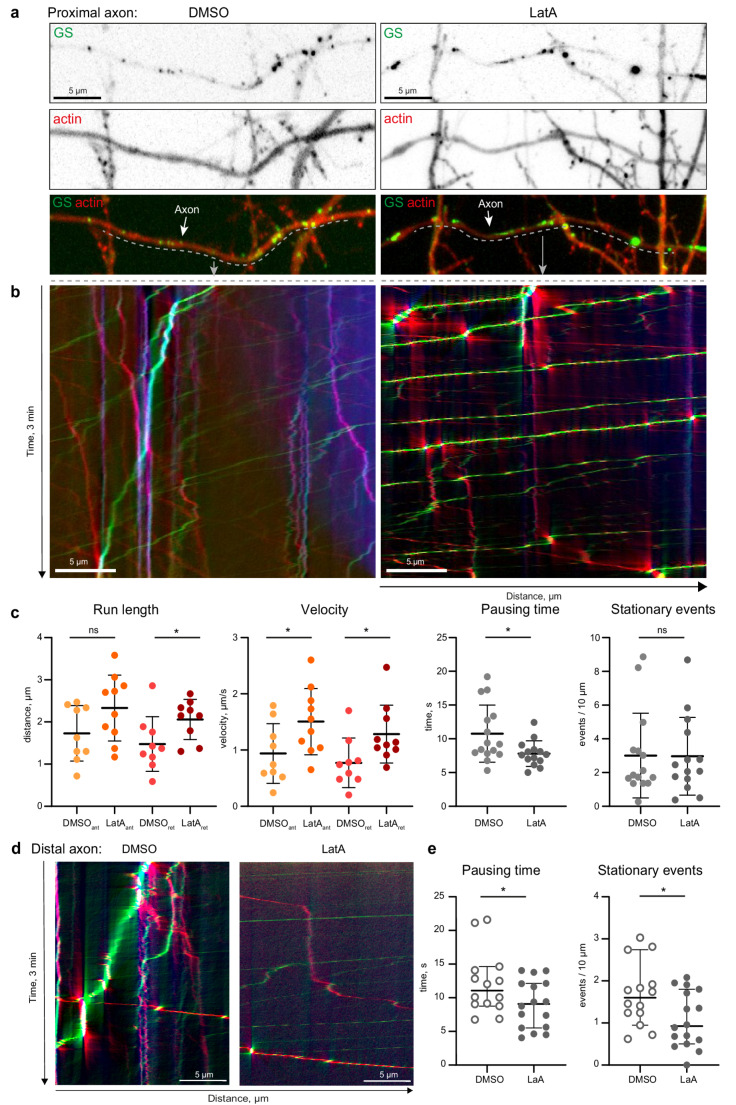
F-actin influences the trafficking of GS in axons. **a**) Representative confocal images of Golgi satellites in proximal axons following latrunculin A (LatA) treatment (5 µM, right) and DMSO control treatment (left) in relation to actin (pORANGE-actin-tagRFP endogenous labelling and St3gal5-GFP overexpression). See also videos 4 (DMSO) and 5 (LatA). **b**) Kymographs of the videos shown in a), recorded for 3 min at 2 FPS, showing increased movement and decreased pausing of the GS after LatA treatment compared to DMSO ctr. **c**) Quantification of GS trafficking parameters in the proximal axon: run length, velocity, pausing time and stationary GS; run length and velocity are increased in the LatA treatment group (p (anterograde run length) = 0.0891, p* (retrograde run length) = 0.0455, p* (anterograde velocity) = 0.0425, p* (retrograde velocity = 0.0334); unpaired t-test; n (DMSO) = 9, n (LatA) = 10; one axon from each cell in 3 individual cultures). The pausing time is significantly reduced by LatA treatment (p* = 0.0240; unpaired t-test; n (DMSO) = 15, n (LatA) = 14; one axon from each cell in 3 individual cultures). All values are presented as mean ± SD. Quantification of stationary events per 10 μm showing no difference in stationary events (*p* = 0.9659; unpaired t-test; n (DMSO) = 15, n (LatA) = 14; one axon from one cell in 3 single cultures), presented as mean ± SD. **d**) Representative kymographs of confocal time-lapse images of GS in proximal axons following 30 min of latrunculin A (LatA, 5 µM, right panel) and DMSO control treatment (left). Videos and Kymographs as described in a) and b). **e**) Quantification of immobile GS in the distal axon: pausing time and stationary events are measured. The pausing time is significantly reduced by LatA treatment (p* = 0,0490; unpaired t-test; n (DMSO) = 14, n (LatA) = 16; one axon from each cell in 3 individual cultures). All values are presented as mean ± SD. Quantification of stationary events per 10 μm being significantly reduced by LatA treatment (p* = 0.0274; unpaired t-test n (DMSO) = 14, n (LatA) = 16; one axon from one cell in 3 single cultures), presented as mean ± SD

The F-actin cytoskeleton is known to regulate the transport and localization of dendritic secretory organelles, including lysosomes and the spine apparatus [20, 25]. Furthermore, F-actin is highly enriched in presynaptic boutons [26]. We therefore asked whether presynaptic F-actin is capable of regulating organelle positioning. To determine the contribution of F-actin to GS recruitment, we co-transfected primary hippocampal neurons with St3gal5-GFP and pORANGE-actin-TagRFP (CRISPR/Cas9-based endogenous actin knock-in label) to visualize GS and endogenous actin in live cells [27]. Various F-actin structures are present in neurons, including a dense F-actin mesh in dendritic spines, F-actin patches in dendrites, F-actin patches at presynaptic sites, longitudinal actin fibers, and a membrane periodic skeleton (MPS), which consists of F-actin, spectrin, and associated proteins in axons, dendrites, and the neck of dendritic spines [28–32]. In our experiment, such local enrichments of F-actin were visible in confocal images as regions exhibiting a higher actin-TagRFP signal intensity (Fig. 5a, left). We then treated the cells with either the F-actin depolymerizing agent latrunculin A (LatA) (5 µM) or a solvent control (DMSO) for 30 min, after which time-lapse imaging was performed (DIV16-17 video 5&6) (Fig. 5a). As expected, the actin-TagRFP labeling appeared smoother and more homogenous following LatA treatment (Fig. 5a, right). Kymographs revealed a drastic effect of F-actin depolymerization on GS behavior (Fig. 5c). We analyzed GS motility in proximal axons because it is easier to identify whether vesicles are moving toward or away from the soma and found that GS became faster in both directions (mean velocity: DMSO-Ant = 0.9 μm/s, LatA-Ant = 1.5 μm/s, DMSO-Ret = 0.8 μm/s, LatA-Ret = 1.3 μm/s), showed longer run lengths (mean run length: DMSO-Ant = 1.7 μm, LatA-Ant = 2.3 μm, DMSO-Ret = 1.5 μm, LatA-Ret = 2.1 μm) and shorter pausing times (mean DMSO = 10.8 s, mean LatA = 7.8 s) after F-actin depolymerization. However, the number of stationary GS was not significantly altered. Since we showed previously that GS stalling occurs preferentially at presynaptic sites, and as synaptic boutons are more abundant in the distal axon, we next compared the stalling behavior in proximal axons to that in distal axons (Fig. 5d). In both compartments, GS paused for a shorter time following LatA treatment (mean DMSO = 12.11 s, mean LatA = 9.03 s). Unlike the proximal axon, the number of stationary GS in the distal axon was significantly reduced (mean DMSO = 1.68, mean LatA = 1.08). This suggests an F-actin-dependent recruitment of GS to pre-synapses in the more distal part of the axon, where they remain anchored for longer periods of time.

### Axonal GS are actively anchored to actin filaments by myosin VI motor protein

**Fig. 6:**
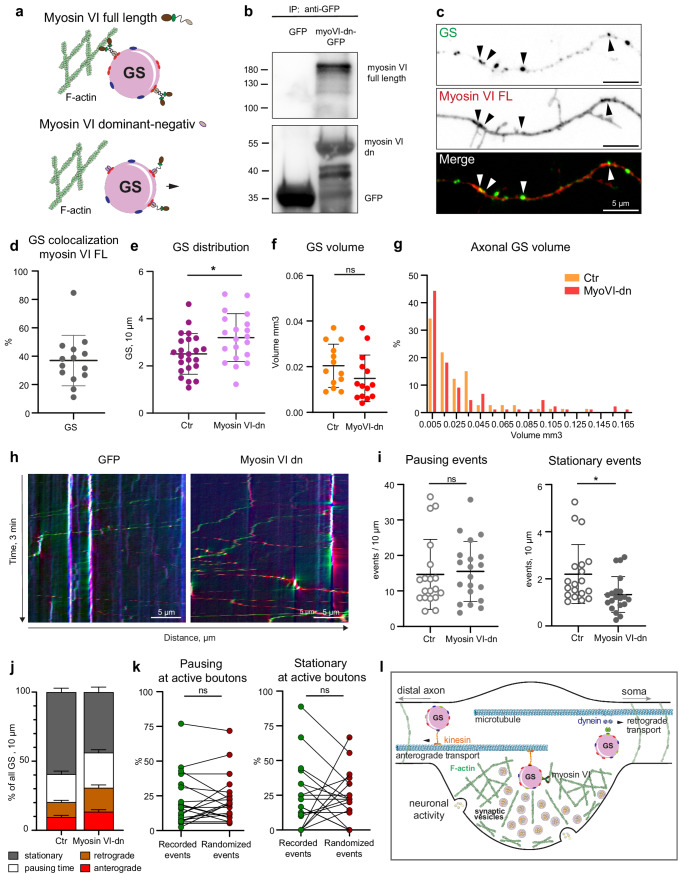
Myosin VI mediates the anchoring of GS in axons. **a**) Schematic of the myosin VI dominant negative (MyoVI-dn) principle. The motor domains of myosin VI are removed, preventing its interaction with actin (created with Servier medical Art templates, Table S1). **b**) Western blot of MyoVI-dn-GFP co-IP from HEK293T cells extract compared to GFP ctr. showing interaction of MyoVI-dn with endogenous myosin detected with a myosin VI antibody (upper panel) and a GFP antibody (lower panel). **c**) Representative image showing the colocalization of MyoVI-FL-GFP and GS (St3gal5-HaloTag-JF646). **d**) Quantification of the colocalization of GS (St3gal5-HaloTag) in axons with MyoVI-full length (MyoVI-full length-GFP) revealing that ~ 40% of GS contain Myosin VI-FL (*n* = 15 axons, one axon from one cell in 2 individual cultures), presented as mean ± SD. **e**) Quantification of the distribution of GS (St3gal5-HaloTag) in axons upon overexpression of MyoVI-dn-GFP compared to a control (GFP overexpression) showing a significant increase in GS per 10 μm upon MyoVI-dn overexpression in medial/distal axons (p* = 0.0206; unpaired t-test; n (Ctr) = 23, n (MyoVI-dn) = 20; one axon from one cell in 3 individual cultures), presented as mean ± SD. **f**) Quantification of the volume of GS in the axon upon MyoVI-dn overexpression showing no significant change in the volume: *p* = 0.1575; t- test; n: (axon-ctr) = 13, (axon-MyoVI-dn) = 14; one axon per cell in two individual cultures; single values represent the median per cell, presented as mean ± SD. **g**) Frequency distribution of individual GS volumes in the axon upon MyoVI-dn overexpression, showing a slight reduction in the smaller GS fractions. Same n as in f, represented as % of GS being in the size range. **h**) Representative kymographs of GS (St3gal5-HaloTag-JF646) in axons showing that overexpression of MyoVI-dn-GFP results in fewer stationary events compared to a GFP control. **i**) Quantification of pausing and stationary events per 10 μm. The pausing events are not altered (*p* = 0.7723), where the stationary events show a significant reduction after MyoVI-dn overexpression (p* = 0.0126; unpaired t-test; n (Ctr) = 19, n (MyoVI-dn) = 20; one axon from one cell in 4 individual cultures), presented as mean ± SD. **j**) Time percentage of GS being stationary, pausing, retrogradely or anterogradely transported. Note a reduction in the pausing time in the MyoVI-dn group. **k**) Quantification of GS pausing (left) and being stationary GS (right) in proximity to active boutons upon overexpression of MyoVI-dn showing no significant association of GS pausing or being stationary events to active boutons. For intrinsic control, GS pausing or being stationary events are compared to random pausing events distributed along the examined axon (see Methods), (p (pausing) = 0.6586, p (stationary) = 0.9506; paired t-test; *n* = 21 axons, from two independent cultures). **l**) Model showing the interplay of GS with F-actin and myosin VI at presynaptic boutons

The long-term positioning of secretory organelles, such as lysosomes, at F-actin patches in dendrites is mediated by the dimeric motor protein myosin V [20]. Myosin V is also known to stall dendritic cargo at the AIS and to redirect it into the dendrite [33]. It does not seem to be involved in cargo transport along the axon. On the other hand, the related motor protein myosin VI has been reported to be present in axons and to play a role in the localization of proteins to the axonal membrane [34, 35]. Interestingly, myosin VI can also be activated by elevated Ca^2+^ levels induced by synaptic activity [36]. We therefore hypothesized that myosin VI might be involved in the activity-dependent stalling of GS in the axon. To address this question, we used a GFP-tagged myosin VI dominant negative (MyoVI-dn) construct [20, 37], in which the motor domain of the myosin VI heavy chain as well as the coil domain has been removed, thereby preventing it from binding to F-actin (Fig. 6a). It retains the cargo binding domain and overexpressed MyoVI-dn will thus compete with endogenous myosin VI for available cargo adapters, and impair its function [38, 39]. Co-immunoprecipitation experiments demonstrated that MyoVI-dn interacts with endogenous myosin VI, likely by binding to the same cargo adaptor protein (Fig. 6b). This would result in the formation of a non-motile myosin VI (Fig. 6a). To investigate if myosin VI is recruited to GS and can be involved in GS trafficking, we co-transfected MyoVI-GFP and St3gal5-Halo into primary hippocampal neurons and visualized the GS with the Halo dye JF646. Confocal imaging in medial and distal axons (Fig. 6c) revealed colocalization of GS with MyoVI-full length-GFP (mean = ~ 40%) (Fig. 6d), indicating that myosin VI can be recruited to the surface of GS. To investigate whether MyoVI-dn expression influences GS trafficking and localization, we initially quantified the density of GS in the medial/distal region of the axon, which revealed that MyoVI-dn overexpression increased the total number of GS (Fig. 6e), while the volume of the GS did not change significantly, also there is a trend showing some decrease upon inhibition of myosin VI (mean Axon: Ctr = 0.020 µm^3^, MyoVI-dn = 0.015 µm^3^) (Fig. 6f-g). Furthermore, time-lapse confocal imaging followed by kymograph analysis (Fig. 6h) demonstrated that although the pausing events of GS were not altered, the number of stationary GS was significantly reduced in the MyoVI-dn group (mean Ctr = 2.2 per 10 μm, mean MyoVI-dn = 1.3 per 10 μm) (Fig. 6i). Overall, the fraction of mobile GS was increased, as stationary GS were decreased in the MyoVI-dn group (Fig. 6j). Finally, we investigated if MyoVI-dn overexpression could prevent the stalling of GS at active boutons, similar to Fig. 4c-d. Indeed, we could demonstrate that upon MyoVI-dn overexpression GS no longer were paused nor were stationary at active boutons (Fig. 6k). This suggests that myosin VI plays a role in the long-term anchoring of GS in axons (Fig. 6l).

## Discussion

In this study, we show that Golgi satellites are present in the proximal, medial, and distal regions of the axon, as well as in the axonal growth cone of primary hippocampal neurons. Similar to dendritic GS, they are marked by the GS markers pGolt and α3-sialyltransferase probes, and contribute to local protein glycosylation. GS are transported bidirectionally, frequently pause at boutons, and are preferentially recruited to active presynaptic sites. Moreover, we show that myosin VI is involved in the long-term stalling of GS at active synapses, thereby providing a mechanism for activity-dependent organelle localization.

St3gal3 and St3gal5, Golgi-localized α3-sialyltransferases responsible for the mature N-glycosylation of glycoproteins, and pGolt are well-established markers for dendritic GS [8–10]. We found that these markers exhibited a high degree of colocalization not only in dendrites but also axons (Mikhaylova et al., 2016 and Fig. 1). In this study, we opted to use St3gal5 as a GS marker, since it shows less membrane labelling and has a better signal-to-noise ratio compared to pGolt. Additionally, St3gal5 is not only highly expressed in the brain, but has also been described to localize in axons, however its function in relation to GS remained uninvestigated [40]. Using the St3gal5 label, we characterized the distribution of GS in the axon and found that they are spread out along the entire axonal length, and can even be found inside the growth cone.

What is the function of GS in axons? In analogy to dendritic GS, axonal GS might be involved in protein processing and recycling. Prior research has shown that these organelles serve as distal glycosylation stations within dendrites [8–10]. GS can modify sugars on newly synthesized glycoproteins as they pass through the secretory pathway, and process surface glycoproteins that have been internalized through the endocytic pathway, ensuring proper glycosylation for both newly synthetized proteins and those recycled from the cell surface. Although it has been shown that core glycosylation on transmembrane proteins is sufficient for transmembrane proteins to be functional and localized to the membrane [11], local GS are especially relevant for activity-dependent remodeling of the surface glycoproteome. Indeed, upon neuronal excitation, dendritic GS enable glycoprotein maturation through sialic acid addition, thereby enhancing the functionality of proteins such as nicotinic receptors (Govind et al., 2021) or PSA-NCAM (Andres-Alonso, et al., 2023). Our metabolic labeling experiments indicated that like dendritic GS, axonal GS are involved to the same extent in mature protein glycosylation. It is highly likely that, similar to dendrites, GS in axons constitute specialized microsecretory compartments for posttranslational modification of secreted and transmembrane proteins, as well as local delivery of proteins and membrane components. Several membrane proteins which undergo mature glycosylation, including NCAM1 and nicotinic receptors, are localized to both axons and dendrites [15, 16, 41] and various RNAs coding for transmembrane proteins have been described to present not only in the dendrite but also in the axon [42, 43]. It is possible that they are not only translated but also modified locally in both axons and dendrites without the need to pass through the somatic Golgi. Further research is required to determine the identities of the proteins that undergo glycosylation in axonal GS, as well as the role of neuronal activity in this process. Furthermore, recently trans-Golgi carriers have been shown to deliver cathepsins to maturing lysosomes in axons of cortical neurons [14]. It is possible that GS may have similar functions, and their role in this process remains to be elucidated.

Being part of the local secretory microsystem in axons and dendrites requires active delivery and recruitment of GS at places of high demand. We therefore set out to characterize the transport of GS in distal axonal compartments, where synaptic density and therefore membrane and protein turnover are high. Overall, the trafficking of GS in axons was comparable to that in dendrites in terms of the velocity and directionality of movement (anterograde vs. retrograde transport). However, the fraction of stationary GS was lower in axons, and axonal GS exhibited a greater overall mobility. In contrast to dendrites, microtubules in the axon have a uniform orientation, with the plus end pointing towards the periphery. Since we observed both anterograde and retrograde transport of GS, we conclude that both plus-end and minus-end directed motors (i.e., kinesins and dynein) are involved in GS trafficking. An intriguing question for future research, which could not be addressed in this study, is the origin of GS. A possible approach would be to use long-term imaging combined with photo-conversion or photo-activation of GS markers. Since Govind et al. (2021) demonstrated that prolonged synaptic activity leads to the fragmentation of the somatic Golgi and an increase in the number of dendritic GS, it is important to investigate whether this also results in an increase of axonal GS. Furthermore, it remains to be determined whether this increase is due to enhanced delivery of GS from the soma or if they are assembled locally through the fusion of Golgi-derived vesicles. To investigate at which specific locations GS are stopped along the axon, we analyzed the stalling behavior of GS and demonstrated that GS frequently pause for short timespans at presynaptic boutons, and preferentially anchor for longer timespans at active synapses. The recruitment of organelles to presynaptic boutons upon neuronal activity has also been shown for other vesicles, including DCV and amphisomes [21, 22], suggesting the existence of a general control mechanism regulating organelle positioning at presynapses.

How can active synapses recruit passing organelles? Since presynaptic sites are enriched in F-actin [26], we hypothesized that the mesh of F-actin might act as a physical trap to stop passing organelles. A similar process has also been described in dendrites, where F-actin hot spots found along the dendrite at the base of spines or excitatory shaft synapses can stop and anchor passing lysosomes [20]. To test whether the F-actin network influences GS trafficking in axons, we treated neurons with LatA to depolymerize F-actin, and observed a dramatic effect on GS motility: they became faster, ran longer distances and paused for shorter amounts of time, clearly indicating that F-actin is able to slow down GS transport [20]. Interestingly, F-actin depolymerization led to a reduction of stationary GS only in the distal, but not in the proximal axon. This may be attributed to the fact that the density of presynaptic boutons is higher in the distal part of the axon. Since LatA causes overall F-actin depolymerization by preventing new polymerization, its effect on different F-actin structures also depends on their intrinsic turnover rates. F-actin in presynaptic boutons has a high turnover rate, and would therefore be more affected by LatA treatment than e.g. the MPS, which has a slower turnover of actin and is more resistant to LatA treatment [44]. It remains to be determined if other specialized F-actin structures, such as the MPS, can serve as organelle docking sites.

Is the stalling of GS at F-actin regulated by a myosin motor protein? Myosin V and VI are processive myosins that are involved in short-range cargo trafficking and localization. Both myosin V and VI have calmodulin binding regions through which they are activated in a calcium-dependent manner [36, 45, 46]. In dendrites, Myosin V is involved in the active retention of lysosomes at F-actin patches [20], and in anchoring the spine apparatus to dendritic spines [25, 47]. In the AIS, it stops mRNA and vesicles containing dendritic proteins such as GluR1 and Kv4.2 to be rerouted towards the dendrite [25, 33, 48, 49]. While no roles have been described for myosin V in more distal parts of the axon apart from the AIS, myosin VI has been shown to be present in the axon, where it is involved in the delivery of proteins to the axonal surface [49]. We therefore investigated a potential role of myosin VI in the axonal trafficking of GS, and found that inhibiting myosin VI activity resulted in an increase in mobile GS. We therefore propose a scenario where GS are recruited to active boutons by myosin VI, which can be locally activated by Ca^2+^ influx triggered by synaptic activity. It is plausible that this mechanism could also apply to other secretory vesicles, enabling their interaction at sites of high protein turnover, such as active synapses. In addition, GS may fuse with each other or with other secretory organelles in a myosin VI-dependent manner, as the fusion of autophagosomes and lysosomes is known to be myosin VI-dependent in non-neuronal cells [50]. A reduced incidence of GS-GS fusion could explain why we observed an increased number of GS in distal axons upon myosin VI inhibition.

In summary, we described the axonal presence and activity-dependent localization of GS at synaptic boutons, which provides new possibilities for understanding and interpretations of mechanisms involved in local synaptic proteostasis.

## Supplementary Information

Below is the link to the electronic supplementary material.

Supplementary File 1 (MP4 14.9 MB) Video 1: GS trafficking in axons of primary hippocampal neurons. Representative video showing GS (St3gal5-GFP in green) and Actin-RFP (red) imaged with 4 Frames per second (FPS) for 2 minutes in dissociated primary rat hippocampal neurons (DIV 16-17).

Supplementary File 2 (MP4 4.59 MB) Video 2: GS trafficking in dendrites of primary hippocampal neurons. Representative video showing (St3gal5-GFP in green) and Actin-RFP (red) imaged with 4 FPS for 2 minutes in dissociated primary rat hippocampal neurons (DIV 16-17).

Supplementary File 3 (MP4 1.59 MB) Video 3: GS pause at active presynaptic boutons. Representative video showing (St3gal5-GFP in green), mRuby (blue) and active boutons (synaptotagmin antibody assay, red) imaged with 2 FPS for 3 minutes in dissociated primary rat hippocampal neurons (DIV 16-17).

Supplementary File 5 (MP4 252 KB) Video 5: GS trafficking in axons, solvent control (DMSO). Representative video showing GS (St3gal5-GFP in green) and endogenous actin (pORANGE-actin- TagRFP; CRISPR/Cas9-based endogenous actin knock-in label in red) imaged with 2 FPS for 3 minutes in dissociated primary rat hippocampal neurons (DIV 16-17).

Supplementary File 6 (MP4 90.6 KB) Video 6: GS trafficking in axons, latrunculin A treatment. Representative video showing GS (St3gal5-GFP in green) and endogenous actin (pORANGE-actin- TagRFP; CRISPR/Cas9-based endogenous actin knock-in label in red) imaged with 2 FPS for 3 minutes in dissociated primary rat hippocampal neurons (DIV 16-17).

Supplementary File 7 (PDF 79.5 KB)

Supplementary File 4 (MP4 40.5 MB) Video 4: GS at boutons with calcium transients. Representative video showing (St3gal5-GFP in green) and the calcium indicator GCaMP6s (magenta) imaged with 10 frames per second for 3 minutes in dissociated primary rat hippocampal neurons (DIV 16-17).

## Data Availability

Materials (plasmids) can be requested from Marina Mikhaylova.

## Abbreviations

AAV9: Adeno-associated viruses 9
AC4GalNAZ: N-azidoacetylgalactosamine-tetraacylated
AC4GlcNAZ: N-azidoacetylglucosamine-tetraacylated
aCSF: artificial cerebrospinal fluid solution
AF488: Alexa Fluor^®^ 488
AIS: axon initial segment
Ant: anterograde
BBHE: blocking buffer horse emulsion
Co-IP: co-immunoprecipitation
CRISPR-Cas9: clustered regularly interspaced short palindromic repeats - CRISPR associated
Ctr.: control
DABCO: triethylenediamine
DCV: dense-core vesicles
DIV: day in vitro
DMEM: Dulbecco’s modified Eagle’s medium
DMSO: dimethyl sulfoxide
EGFP: enhanced green fluorescent protein
ER: endoplasmic reticulum
ERES: ER exit-sites
ERGIC: endoplasmic-reticulum-Golgi intermediate compartment
F-actin: filamentous actin
FCS: fetal calf serum
FPS: frames per second
FRAP: fluorescence recovery after photobleaching
GFP: green fluorescent Protein
GO: Golgi outposts
GS: Golgi satellites
HBSS: Hanks’ Balanced Salt Solution
HEK293T: human embryonic kidney 293T
ICC: immunocytochemistry
JF646: Janelia Fluor^®^ 646
LatA: latrunculin A
MAP2: microtubule-associated protein 2
MPS: membrane periodic skeleton
MT: microtubule
MyoVI-dn: myosin VI dominant-negative
MyoVI-FL: myosin VI full length
NCAM: neural cell adhesion molecule
PBS: phosphate-buffered saline
PCR: polymerase chain reaction
PFA: paraformaldehyde
PSA-NCAM: polysialylated-neural cell adhesion molecule
Ret: retrograde
RFP: red fluorescent protein
ROI: region of interest
SD: standard deviation
SDS-PAGE: sodium dodecyl sulfate-polyacrylamide gel electrophoresis
SN: supernatant
St3gal5: ST3 β-galactoside α-2,3-sialyltransferase 5
TIRF: total internal reflection fluorescence
TRPM8: transient receptor potential cation channel subfamily M (melastatin) member 8

## References

[1] Pará C, Bose P, Pshezhetsky AV (2020) Neuropathophysiology of lysosomal storage diseases: synaptic dysfunction as a starting point for disease progression. J Clin Med 9(3):616. 10.3390/jcm9030616 32106459 10.3390/jcm9030616 PMC7141115

[2] Wang X, Huang T, Bu G, Xu H (2014) Dysregulation of protein trafficking in neurodegeneration. Mol Neurodegener 9:31 25152012 10.1186/1750-1326-9-31 PMC4237948

[3] Hanus C, Ehlers MD (2008) Secretory outposts for the local processing of membrane cargo in neuronal dendrites. Traffic 9:1437–1445 18532987 10.1111/j.1600-0854.2008.00775.x PMC2572994

[4] Ori-McKenney KM, Jan LY, Jan YN (2012) Golgi outposts shape dendrite morphology by functioning as sites of acentrosomal microtubule nucleation in neurons. Neuron 76:921–930. 10.1016/j.neuron.2012.10.008 23217741 10.1016/j.neuron.2012.10.008 PMC3523279

[5] Horton AC, Rácz B, Monson EE et al (2005) Polarized secretory trafficking directs cargo for asymmetric dendrite growth and morphogenesis. Neuron 48:757–771. 10.1016/j.neuron.2005.11.005 16337914 10.1016/j.neuron.2005.11.005

[6] Kemal S, Richardson HS, Dyne ED, Fu MM (2022) ER and golgi trafficking in axons, dendrites, and glial processes. Curr Opin Cell Biol 78:102119. 10.1016/j.ceb.2022.102119 10.1016/j.ceb.2022.102119 PMC9590103 35964523

[7] Wang T, Hanus C, Cui T et al (2012) Local zones of endoplasmic reticulum complexity confine cargo in neuronal dendrites. Cell 148:309–321. 10.1016/j.cell.2011.11.056 22265418 10.1016/j.cell.2011.11.056 PMC3266556

[8] Mikhaylova M, Bera S, Kobler O et al (2016) A dendritic golgi satellite between ERGIC and retromer. Cell Rep 14:189–199. 10.1016/j.celrep.2015.12.024 26748700 10.1016/j.celrep.2015.12.024

[9] Andres-Alonso M, Borgmeyer M, Mirzapourdelavar H et al (2023) Golgi satellites are essential for polysialylation of NCAM and expression of LTP at distal synapses. Cell Rep. 10.1016/j.celrep.2023.112692 37355986 10.1016/j.celrep.2023.112692

[10] Govind AP, Jeyifous O, Russell TA et al (2021) Activity-dependent golgi satellite formation in dendrites reshapes the neuronal surface glycoproteome. Elife 10:68910. 10.7554/eLife 10.7554/eLife.68910 PMC8494481 34545811

[11] Hanus C, Geptin H, Tushev G et al (2016) Unconventional secretory processing diversifies neuronal ion channel properties. Elife 5:. 10.7554/eLife.20609.001. 10.7554/eLife.20609 PMC5077297 27677849

[12] Cornejo VH, González C, Campos M et al (2020) Non-conventional axonal organelles control TRPM8 ion channel trafficking and peripheral cold sensing. Cell Rep 30:4505–4517e5. 10.1016/j.celrep.2020.03.017 32234483 10.1016/j.celrep.2020.03.017

[13] González C, Cánovas J, Fresno J et al (2016) Axons provide the secretory machinery for trafficking of voltage-gated sodium channels in peripheral nerve. Proc Natl Acad Sci U S A 113:1823–1828. 10.1073/pnas.1514943113 26839409 10.1073/pnas.1514943113 PMC4763731

[14] Lie PPY, Yang DS, Stavrides P et al (2021) Post-golgi carriers, not lysosomes, confer lysosomal properties to pre-degradative organelles in normal and dystrophic axons. Cell Rep. 10.1016/j.celrep.2021.109034 33910020 10.1016/j.celrep.2021.109034 PMC8135226

[15] Doherty P, Cohen J, Walsh FS (1990) Neurite outgrowth in response to transfected N-CAM changes during development and is modulated by polysialic acid. Neuron 5:209–219. 10.1016/0896-6273(90)90310-c 2200449 10.1016/0896-6273(90)90310-c

[16] Muller D, Stoppini L, Wang C, Kiss JZ (1994) A role for polysialylated neural cell adhesion molcule in lesion-induced sprouting in hippocampal organotypic cultures. Neuroscience 61:441–445. 10.1016/0306-4522(94)90424-3 7969921 10.1016/0306-4522(94)90424-3

[17] Kapitein LC, Yau KW, Hoogenraad CC (2010) Microtubule dynamics in dendritic spines. Methods Cell Biol 97:111–132. 10.1016/S0091-679X(10)97007-6 20719268 10.1016/S0091-679X(10)97007-6

[18] Mangeol P, Prevo B, Peterman EJG (2016) Kymographclear and kymographdirect: two tools for the automated quantitative analysis of molecular and cellular dynamics using kymographs. Mol Biol Cell 27:1948–1957. 10.1091/mbc.E15-06-0404 27099372 10.1091/mbc.E15-06-0404 PMC4907728

[19] Tinevez JY, Perry N, Schindelin J et al (2017) TrackMate: an open and extensible platform for single-particle tracking. Methods 115:80–90. 10.1016/j.ymeth.2016.09.016 27713081 10.1016/j.ymeth.2016.09.016

[20] van Bommel B, Konietzny A, Kobler O et al (2019) F-actin patches associated with glutamatergic synapses control positioning of dendritic lysosomes. EMBO J. 10.15252/embj.2018101183 31267565 10.15252/embj.2018101183 PMC6669925

[21] Nassal JP, Murphy FH, Toonen RF, Verhage M (2022) Differential axonal trafficking of neuropeptide Y-, LAMP1-, and RAB7-tagged organelles in vivo. Elife. 10.7554/ELIFE.81721 36459486 10.7554/eLife.81721 PMC9718525

[22] Andres-Alonso M, Ammar MR, Butnaru I et al (2019) SIPA1L2 controls trafficking and local signaling of TrkB-containing amphisomes at presynaptic terminals. Nat Commun. 10.1038/s41467-019-13224-z 31784514 10.1038/s41467-019-13224-z PMC6884526

[23] Qian P, Manubens-Gil L, Jiang S, Peng H (2024) Non-homogenous axonal bouton distribution in whole-brain single-cell neuronal networks. Cell Rep. 10.1016/j.celrep.2024.113871 38451816 10.1016/j.celrep.2024.113871

[24] Guedes-Dias P, Nirschl JJ, Abreu N et al (2019) Kinesin-3 responds to local microtubule dynamics to target synaptic cargo delivery to the presynapse. Curr Biol 29:268–282e8. 10.1016/j.cub.2018.11.065 30612907 10.1016/j.cub.2018.11.065 PMC6342647

[25] Konietzny A, Grendel J, Kadek A et al (2022) Caldendrin and myosin V regulate synaptic spine apparatus localization via ER stabilization in dendritic spines. EMBO J. 10.15252/embj.2020106523 34935159 10.15252/embj.2020106523 PMC8844991

[26] Bingham D, Jakobs CE, Wernert F et al (2023) Presynapses contain distinct actin nanostructures. J Cell Biol. 10.1083/jcb.202208110 37578754 10.1083/jcb.202208110 PMC10424573

[27] Willems J, de Jong APH, Scheefhals N et al (2020) Orange: a CRISPR/Cas9-based genome editing toolbox for epitope tagging of endogenous proteins in neurons. PLoS Biol. 10.1371/journal.pbio.3000665 32275651 10.1371/journal.pbio.3000665 PMC7176289

[28] Konietzny A, Bär J, Mikhaylova M (2017) Dendritic actin cytoskeleton: structure, functions, and regulations. Front Cell Neurosci. 10.3389/fncel.2017.00147. 11:28572759 10.3389/fncel.2017.00147 PMC5435805

[29] Kevenaar JT, Hoogenraad CC (2015) The axonal cytoskeleton: from organization to function. Front Mol Neurosci 8:44. 10.3389/fnmol.2015.00044 26321907 10.3389/fnmol.2015.00044 PMC4536388

[30] Xu K, Zhong G, Zhuang X (2013) Actin, spectrin, and associated proteins form a periodic cytoskeletal structure in axons. Science 339:452–456. 10.1126/science.1232251 23239625 10.1126/science.1232251 PMC3815867

[31] Bär J, Kobler O, Van Bommel B, Mikhaylova M (2016) Periodic F-actin structures shape the neck of dendritic spines. Sci Rep. 10.1038/srep37136 27841352 10.1038/srep37136 PMC5107894

[32] D’Este E, Kamin D, Göttfert F et al (2015) STED nanoscopy reveals the ubiquity of subcortical cytoskeleton periodicity in living neurons. Cell Rep 10:1246–1251. 10.1016/j.celrep.2015.02.007 25732815 10.1016/j.celrep.2015.02.007

[33] Janssen AFJ, Tas RP, Van Bergeijk P et al (2017) Myosin-V induces cargo immobilization and clustering at the axon initial segment. Front Cell Neurosci. 10.3389/fncel.2017.00260 28894417 10.3389/fncel.2017.00260 PMC5581344

[34] Lewis TL, Mao T, Arnold DB (2011) A role for myosin VI in the localization of axonal proteins. PLoS Biol. 10.1371/journal.pbio.1001021 21390300 10.1371/journal.pbio.1001021 PMC3046960

[35] Wagner W, Lippmann K, Heisler FF et al (2019) Myosin VI drives Clathrin-Mediated AMPA receptor endocytosis to facilitate cerebellar Long-Term depression. Cell Rep 28:11–20e9. 10.1016/j.celrep.2019.06.005 31269433 10.1016/j.celrep.2019.06.005

[36] Batters C, Brack D, Ellrich H et al (2016) Calcium can mobilize and activate myosin-VI. Proc Natl Acad Sci U S A 113:E1162–E1169. 10.1073/pnas.1519435113 26811464 10.1073/pnas.1519435113 PMC4780617

[37] Correia SS, Bassani S, Brown TC et al (2008) Motor protein-dependent transport of AMPA receptors into spines during long-term potentiation. Nat Neurosci 11:457–466. 10.1038/nn2063 18311135 10.1038/nn2063

[38] Phichith D, Travaglia M, Yang Z et al (2009) Cargo binding induces dimerization of myosin VI. Proc Natl Acad Sci U S A 106:17320–17324. 10.1073/pnas.0909748106 19805065 10.1073/pnas.0909748106 PMC2753641

[39] Hu S, Guo Y, Wang Y et al (2019) Structure of myosin VI/Tom1 complex reveals a cargo recognition mode of myosin VI for tethering. Nat Commun 10:1–12. 10.1038/s41467-019-11481-6 31371777 10.1038/s41467-019-11481-6 PMC6673701

[40] Stern CA, Tiemeyer M (2001) A ganglioside-specific sialyltransferase localizes to axons and non-Golgi structures in neurons. J Neuroscienc 21:1434–1443. 10.1523/JNEUROSCI.21-05-01434.2001 10.1523/JNEUROSCI.21-05-01434.2001 PMC6762956 11222633

[41] Role LW, Berg DK (1996) Nicotinic Receptors Review in the Development and Modulation of CNS Synapses. Sargent 10.1016/s0896-6273(00)80134-8 8663984

[42] Hafner AS, Donlin-Asp PG, Leitch B, Herzog E, Schuman EM (2019) Local protein synthesis is a ubiquitous feature of neuronal pre-and postsynaptic compartments. Science 364(6441):eaau3644. 10.1126/science.aau3644 10.1126/science.aau3644 31097639

[43] Shigeoka T, Jung H, Jung J et al (2016) Dynamic axonal translation in developing and mature visual circuits. Cell 166:181–192. 10.1016/j.cell.2016.05.029 27321671 10.1016/j.cell.2016.05.029 PMC4930487

[44] Abouelezz A, Micinski D, Lipponen A, Hotulainen P (2019) Sub-membranous actin rings in the axon initial segment are resistant to the action of latrunculin. Biol Chem 400:1141–1146. 10.1515/hsz-2019-0111 30951495 10.1515/hsz-2019-0111

[45] Shen M, Zhang N, Zheng S et al (2016) Calmodulin in complex with the first IQ motif of myosin-5a functions as an intact calcium sensor. Proc Natl Acad Sci U S A 113:E5812–E5820. 10.1073/pnas.1607702113 27647889 10.1073/pnas.1607702113 PMC5056106

[46] Sellers JR, Thirumurugan K, Sakamoto T et al (2008) Calcium and cargoes as regulators of myosin 5a activity. Biochem Biophys Res Commun 369:176–181. 10.1016/j.bbrc.2007.11.109 18060865 10.1016/j.bbrc.2007.11.109

[47] Konietzny A, González-Gallego J, Bär J et al (2019) Myosin V regulates synaptopodin clustering and localization in the dendrites of hippocampal neurons. J Cell Sci. 10.1242/jcs.230177 31371487 10.1242/jcs.230177 PMC6737913

[48] Balasanyan V, Arnold DB, Baudry M (2014) Actin and Myosin-Dependent localization of mRNA to dendrites. PLoS ONE 9. 10.1371/journal.pone 10.1371/journal.pone.0092349 PMC3956895 24637809

[49] Lewis TL, Mao T, Svoboda K, Arnold DB (2009) Myosin-dependent targeting of transmembrane proteins to neuronal dendrites. Nat Neurosci 12:568–576. 10.1038/nn.2318 19377470 10.1038/nn.2318 PMC2937175

[50] Tumbarello DA, Waxse BJ, Arden SD et al (2012) Autophagy receptors link myosin VI to autophagosomes to mediate Tom1-dependent autophagosome maturation and fusion with the lysosome. Nat Cell Biol 14:1024–1035. 10.1038/ncb2589 23023224 10.1038/ncb2589 PMC3472162

