# Supplementary material for: Myosin VI controls localization of golgi satellites at active presynaptic boutons
Source: Cell Mol Life Sci. 2025 Oct 21;82(1):357. doi: 10.1007/s00018-025-05896-2 (PMC12540243; doi:10.1007/s00018-025-05896-2)
Supplement: Supplementary file 7 — Supplementary File 4 (MP4 40.5 MB) Video 4: GS at boutons with calcium transients. Representative video showing (St3gal5-GFP in green) and the calcium indicator GCaMP6s (magenta) imaged with 10 frames per second for 3 minutes in dissociated primary rat hippocampal neurons (DIV 16-17). [file 18_2025_5896_MOESM7_ESM.pdf]

Supplemental Table 1

REAGENT or RESOURCE	Source	Application and Dilution	Identifier
**Antibodies**			
anti-AnkyrinG (ms)	*EMD Millipore*	1:500	*clone N106/36*
anti-GFP-Atto-488 (camelid sdAb)	NanoTag	1:500-1:250	Clone 1H1/1B2
anti-bassoon (gp)	SySy	1:1000	Lot: 141004
Anti-MAP2 (ms)	Sigma	1:500	clone HM-2
Anti-MAP2 (chick)	Synaptic Systems	1:500	Charge 1-13
Anti-synaptotagmin1-Oyster-650 (ms)	sysy	1:100	105311C5
anti mouse HRP	Jackson ImmunoResearch	1:10000	RRID:AB_2340296
anti-chicken igY H&L (goat)-Alexa-Fluor-405	Abcam	1:500	Lot: GR3291041-3
anti-guinea pig (goat)-Alexa488	Invitrogen	1:500	Lot: 2087691
anti-guinea pig IgG H&L (goat)-Alexa-405	Abcam	1:500	Lot: GR3440099-1
anti-guinea pig(goat)-Abberior Star 635	Abberior	1:500	Lot: 18032019HP
anti-guinea pig(goat)-Alexa568Invitrogen	Invitrogen	1:500	A11075
anti-mouse (goat)-abberior Star 580	Abberior	1:500	LOT 20510PK-1
anti-mouse (goat)-Alexa-405	Abcam	1:500	GR3204627-3
anti-mouse (goat)-Alexa-568	Life Technologies	1:500	Lot: 2216598
Anti-mouse (goat)-Alexa647	Life Technologies	1:500	Lot: 2229182
**Bacterial Strains**			
E. coli XL10Gold	Agilent		Cat#200314
**Chemicals, Pharmalogicals**			
Latrunculine	Tocris	5 µM	3973
DBCO-AF488	Jena Biosciences	1µm	CLK-1278-1
Ac4GalNAz	Jena Biosciences	1µm	CLK-1086-5
Ac4GlcNAz	Jena Biosciences	1µm	CLK-1085-5
Halo Tag Dye JF 646	Promega	200 nM	GA112A
Lipofectamine 2000	Invitrogen	1:2 (DNA:Lipo)	Cat#11668027
**Critical Commercial Assays**			
Anti-GFP-beads	ChromoTek		gtma
pico WB detection	Thermo Scientific™		34580
**Experimental Models: Cell Lines**			
HEK293T	DSMZ		#ACC635

Experimental Models: Organisms/Strains			
Rat: Wistar Unilever	Envigo		HsdCpb:WU

Software and Algorithms			
(Fiji is just) ImageJ Versions 1.49v & 1.53b			http://fiji.sc/; RRID:SCR_002285
Servier medical Art template			licensed under a Creative Commons Attribution 4.0 Unported Licencse; https://smart.servier.com
Prism v.9.1	GraphPad		https://www.graphpad.com/scientific-software/prism/

DNA constructs			
**Backbone**	**Promoter**	**Insert**	**Source**
pAAV	synapsin	empty	gift from Thomas Oertner
pAAV	synapsin	St3ga5l-GFP	This study insert from (Mikhaylova et.al 2016)
pGFP-C1	CMV	GFP	Clontech
mEmerald	CMV	Myosin VI Full-length-pmEmerald	This study, insert from Wolfgang Wagner
mEmerald	CMV	Myosin VI C-terminal domain (dominant negative)	(van Bommel et. al 2019)
pAAV	synapsin	GFP-Myosin VI C-terminal domain; CTD (3177-3789) dominant negative	(van Bommel et. al 2019)
pAAV	synapsin	GCaMP6s	gift from Thomas Oertner
pORANGE	U6	pOrange Actb KI #2-TagRFP	This study
pAAV	synapsin	PSD95-FingR-mRuby2-DTE	This study
pAAV	synapsin	mRuby3	gift from Thomas Oertner
pAAV	synapsin	St3ga5l-Halo	This study
pAAV	beta-actin	actin-mRFP	(van Bommel et. al 2019)
pAAV	synapsin	pGolt-mCherry	Mikhaylova et. al 2016
